# Supplementary figures and images for: Development of a prognostic RiskScore model using efferocytosis-related signature genes for lung adenocarcinoma
Source: PeerJ. 2025 Sep 5;13:e19892. doi: 10.7717/peerj.19892 (PMC12422274; doi:10.7717/peerj.19892)

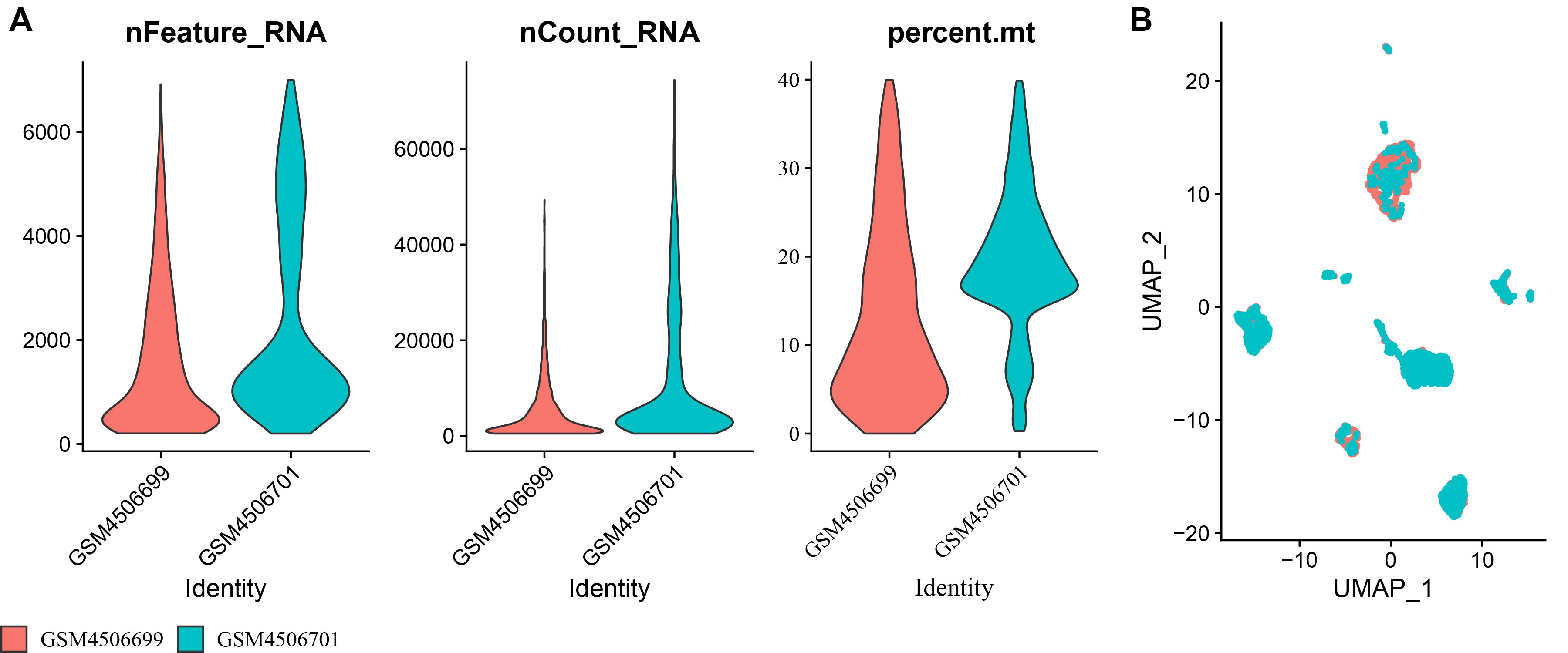

Supplement: Supplemental Information 1 — (A) The nFeature_RNA, nCount_RNA, and percent.mt of LUAD samples; (B) UMAP plot of LUAD samples. [file peerj-13-19892-s001.png]
